# A Phytol-Modified Carbon Veil Paste DNA Sensor for Anticancer Drug Detection

**DOI:** 10.3390/bios16100548

**Published:** 2026-09-30

**Authors:** Kamila Karaguzina, Dmitry Stoikov, Alexey Ivanov, Dominika Kappo, Alan Akhmedov, Rustem Gamirov, Dmitry Shurpik, Ivan Stoikov

**Affiliations:** Alexander Butlerov Institute of Chemistry, Kazan Federal University, 18 Kremlevskaya Street, Kazan 420008, Russia; karkaraguzina@kpfu.ru (K.K.); dkappo@kpfu.ru (D.K.); aaakhmedov@kpfu.ru (A.A.); rusrgamirov@kpfu.ru (R.G.); ivan.stoikov@kpfu.ru (I.S.)

**Keywords:** DNA sensor, amphiphilic phytol derivative, carbon veil, doxorubicin, dacarbazine, carbon veil paste electrode

## Abstract

Electrochemical DNA sensors offer a rapid and miniaturizable approach to monitoring drugs that interact with nucleic acids, but their performance depends on the electrode matrix and DNA immobilization strategy. Here, a renewable bulk-modified carbon veil paste DNA sensor was developed by synergistically combining a custom 3D-printed screw-driven housing and a phytol-derived dicationic amphiphile to retain DNA within a conductive fibrous matrix. Carbon veil paste electrode composition and interfacial properties were studied by voltammetry, electrochemical impedance spectroscopy, and scanning electron microscopy, and analytical performance was evaluated for doxorubicin and dacarbazine in buffer, synthetic plasma, and synthetic urine. The carbon veil matrix provided an effective electroactive area 5.06 times greater than its geometric area. Among the tested modifiers, the phytol-derived amphiphile produced the highest and most stable marker currents. Doxorubicin decreased, whereas dacarbazine increased, the ferrocyanide marker current. Responses were linear over the tested concentration ranges of 1 pM–0.1 µM for doxorubicin and 8 nM–1 µM for dacarbazine, and the determined detection limits were 19 fM and 8.0 nM, respectively. Recoveries in synthetic matrices were 94–99%. The platform provides a renewable DNA-containing electrode architecture for sensitive analysis of individual DNA-interacting drugs in model samples.

## 1. Introduction

Advances in personalized medicine are increasing the demand for rapid methods to monitor drugs in biological fluids. This task is especially relevant for antitumor agents with a narrow therapeutic window, because deviations in their concentration may be accompanied by severe side effects such as cardiotoxicity and bone-marrow suppression [1]. Therapeutic drug monitoring (TDM) of agents like doxorubicin typically targets peak plasma concentrations in the micromolar range, but their prolonged terminal elimination phase causes plasma levels to drop dramatically to the nanomolar and sub-nanomolar ranges [2]. Furthermore, there is a growing demand for ultrasensitive analytical tools in occupational safety to detect trace-level contamination (e.g., via surface wipe samples) in oncology settings [3]. Chromatographic methods, including HPLC and LC-MS, provide high sensitivity and selectivity for such analyses [4]; however, complex sample preparation and the need for centralized instrumentation limit their use for rapid monitoring [5]. Thus, analytical platforms combining broad dynamic ranges with ultra-low detection limits are urgently needed.

Electrochemical biosensors are a promising alternative, offering a fast response, ease of operation, and the possibility of miniaturization [6]. In DNA sensors, immobilized nucleic acids act as the sensing layer, and their interaction with the analyte changes the electrochemical characteristics of the interface [7,8]. In the determination of antitumor drugs, DNA serves simultaneously as a bioreceptor and as a biologically relevant molecular target [9]. Binding of drug molecules to the double helix may involve electrostatic interaction with the phosphate backbone, intercalation, and groove binding [10]. Recording the resulting changes allows the drug concentration to be determined and the features of its interaction with the DNA-containing layer to be assessed [11].

The performance of a DNA sensor is largely governed by the properties of the electrode matrix, which provides charge transfer, analyte access to the sensing layer, and sufficient biomolecule loading. Carbon pastes are of particular interest because they allow bulk modification: functional components are introduced when the carbon powder is mixed with the binder and are distributed throughout the volume of the electrode material [12]. An additional advantage is mechanical renewal of the working surface [13].

Different carbon materials have been used as electrode components in electrochemical biosensors. Carbon nanotubes and graphene improve electrical conductivity and increase the electroactive surface area [14,15]. Porous carbon materials provide a large surface for bioreceptor loading [16], whereas carbon quantum dots contain functional groups that facilitate biomolecule immobilization [17]. However, these particulate modifiers can aggregate and form nonuniform conductive networks, making sensor performance dependent on their dispersion and interparticle contacts [18,19]. Carbon veil differs from these materials because it is already organized as an interconnected network of conductive microfibers with open interfiber pores [20]. Its well-developed fibrous structure and electrical conductivity have led to its use in electrochemical sensors [21,22,23]. Within a paste electrode, such a structure can increase the connectivity of the conductive phase and enlarge the surface available for forming the bioreceptor layer.

Another important task is the stable immobilization of DNA. Its interaction with the carbon surface depends on the material composition [24], the pH and ionic strength of the medium [25], and the contributions of electrostatic, hydrophobic, and π-π interactions [26]. Negatively charged oxygen-containing groups may electrostatically repel the polyanionic DNA backbone [27], which motivates interest in cationic modifiers. Amphiphilic compounds are especially promising: their lipophilic part is retained in the organic binder of the paste, whereas the cationic groups remain available for interaction with DNA [28].

Terpenes and terpenoid derivatives are structurally diverse natural compounds that combine lipophilicity with the possibility of directed functionalization; they can serve as the hydrophobic fragments of such modifiers [29,30]. This makes them promising building blocks for cationic amphiphiles that can anchor in the electrode matrix and interact with negatively charged DNA [31].

Thus, combining carbon veil as the structural basis of the conductive phase with lipophilic cationic amphiphiles as DNA-anchoring agents represents a promising approach to constructing bulk carbon-paste DNA sensors. In this work, we propose a composite paste DNA sensor platform that synergistically combines a custom 3D-printed screw-driven electrode housing for precise surface renewal, carbon veil to form a three-dimensional conductive structure, and synthesized dicationic amphiphiles bearing terpene or octyl hydrophobic fragments to modify the electrode matrix and to anchor DNA. The influence of the modifier structure on the electrochemical characteristics of the platform was studied by voltammetry and electrochemical impedance spectroscopy. The analytical capabilities of the sensor were demonstrated by determining doxorubicin and dacarbazine in model blood-plasma and urine samples. The combination of femtomolar sensitivity toward doxorubicin with a mechanism-dependent, oppositely directed response to the two drugs demonstrates the potential of the platform both for ultrasensitive analysis and for the electrochemical study of drug–DNA interactions.

## 2. Materials and Methods

### 2.1. Reagents

The carbon veil used in this work was produced by Composite Polymer (Moscow, Russia). Double-stranded salmon sperm DNA (Sigma-Aldrich, St. Louis, MO, USA) with an average length of approximately 2000 base pairs was used. The liquid paraffin, doxorubicin hydrochloride, dacarbazine, and HEPES were purchased from Sigma-Aldrich (St. Louis, MO, USA). All other reagents were of analytical grade or higher and were purchased from Sigma-Aldrich (St. Louis, MO, USA). Solutions were prepared with Milli-Q deionized water (Simplicity purification system, Merck-Millipore, Molsheim, France). As the conductive matrix of the biosensor, the carbon veil was first ground with a high-speed electric knife micro-mill (IKA-Werke GmbH & Co. KG, Staufen, Germany) fitted with a stainless-steel chamber to obtain a polydisperse fiber mass. The use of stainless steel allowed efficient grinding of the fibrous material while avoiding absorption into porous surfaces and cross-contamination.

**Figure 1:**
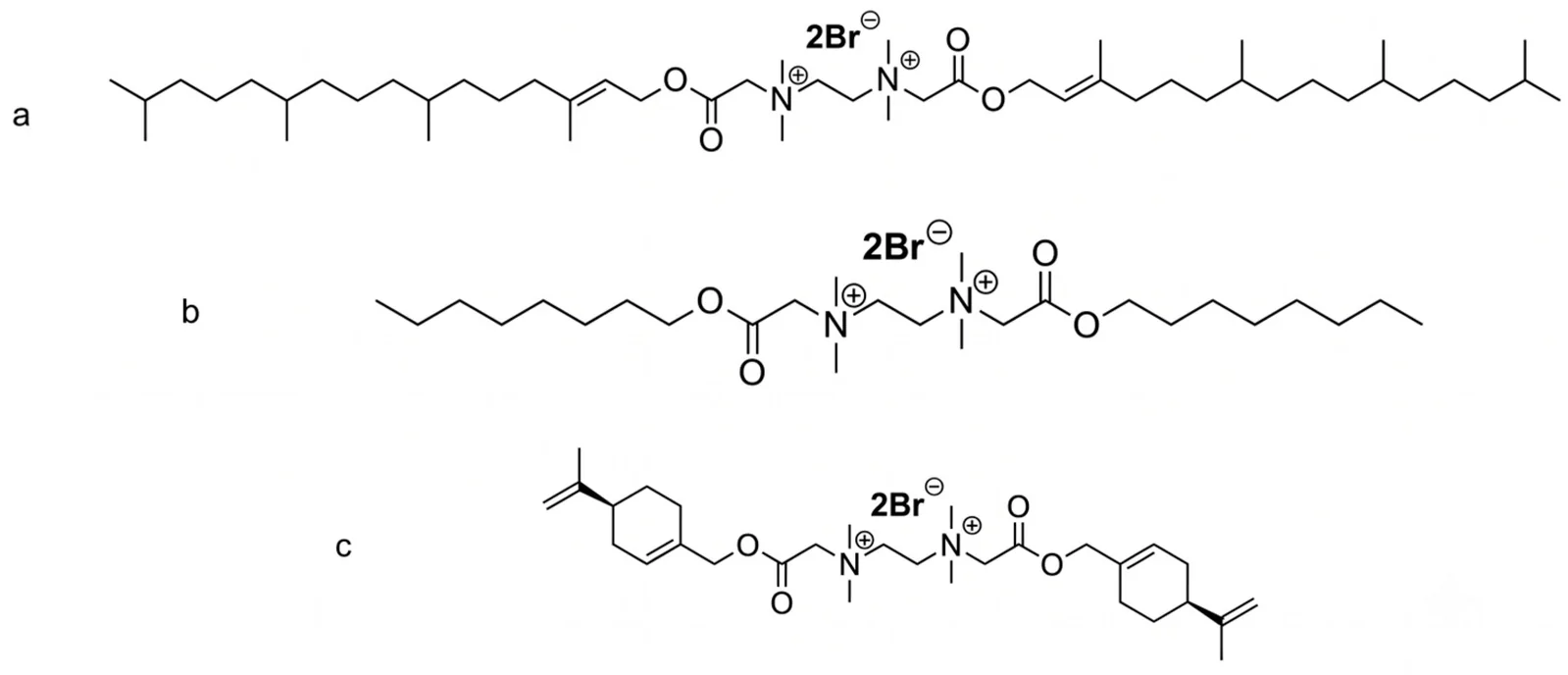
Chemical structures of (**a**) DiPhytTMEDA, (**b**) DiOctTMEDA, and (**c**) DiPerTMEDA.

*N*^1^,*N*^1^,*N*^2^,*N*^2^-Tetramethyl-*N*^1^,*N*^2^-bis(2-oxo-2-((phytyl)oxy)ethyl)ethane-1,2-diammonium bromide (DiPhytTMEDA), *N*^1^,*N*^1^,*N*^2^,*N*^2^-tetramethyl-*N*^1^,*N*^2^-bis(2-oxo-2-((octyl)oxy)ethyl)ethane-1,2-diammonium bromide (DiOctTMEDA), and *N*^1^,*N*^1^,*N*^2^,*N*^2^-tetramethyl-*N*^1^,*N*^2^-bis(2-oxo-2-((perillyl)oxy)ethyl)ethane-1,2-diammonium bromide (DiPerTMEDA) (Figure 1) were synthesized according to our previously reported procedure [32].

### 2.2. Fabrication of the Bulk-Modified Carbon Veil Paste Electrode

**Figure 2:**
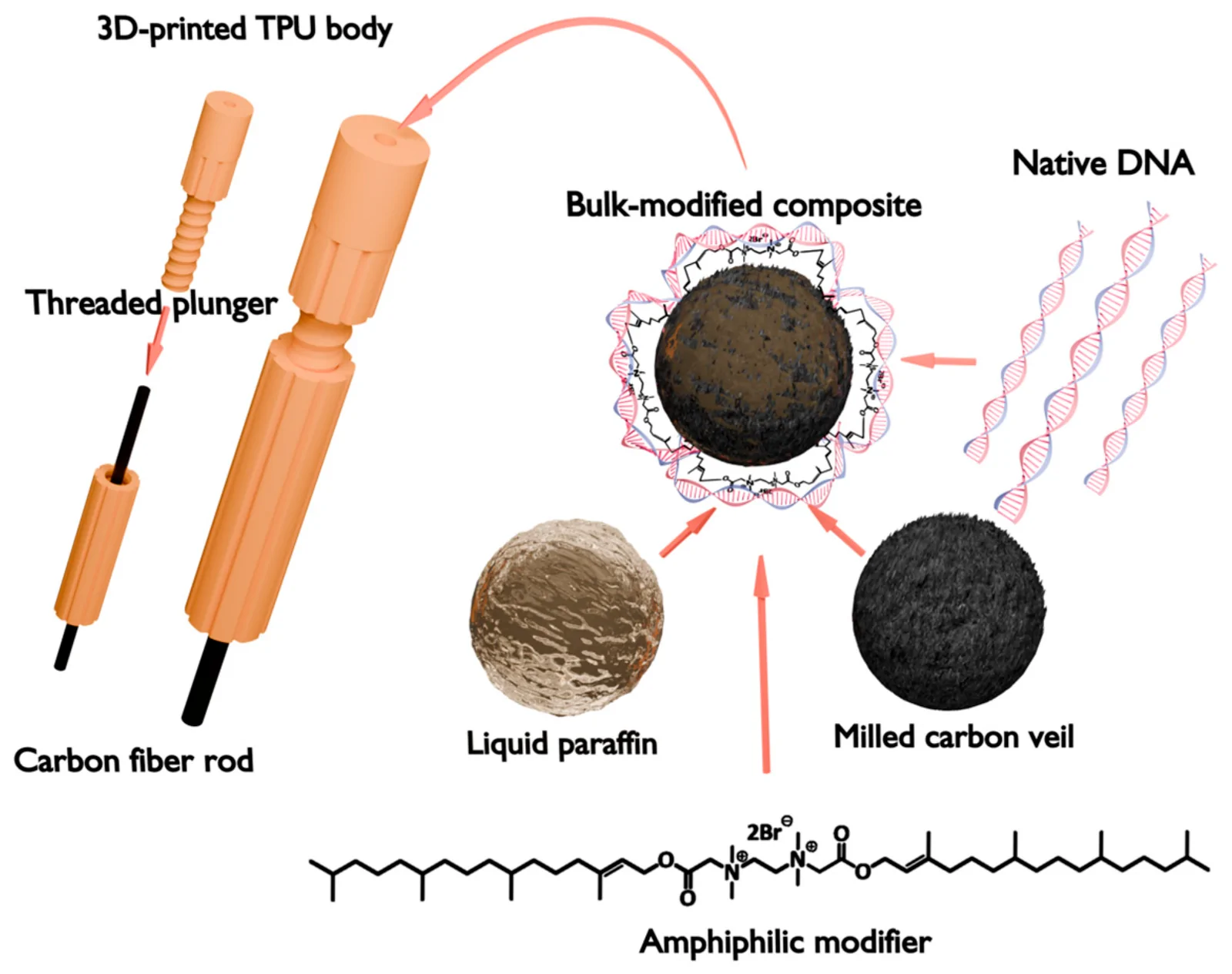
Schematic representation of the 3D-printed CVPE and the composition of the bulk-modified sensing composite based on milled carbon veil, liquid paraffin, DiPhytTMEDA, and DNA. The base carbon paste was prepared by thoroughly mixing ground carbon veil and liquid paraffin at a 1:1 mass ratio. Pink arrows indicate assembly of the electrode and incorporation of the components into the sensing composite.

The CVPE body was fabricated by fused deposition modeling (FDM) from thermoplastic polyurethane (TPU, Bestfilament, Moscow, Russia) using a Flying Bear S1 3D printer (Flying Bear, Jinhua, China) (Figure 2). The choice of this elastomer is justified by its high chemical inertness toward aqueous buffer electrolytes and the organic components of the composite (paraffin), as well as by its specific physico-mechanical properties. The high elasticity and wear resistance of TPU ensured tight sealing of the internal cylindrical cavity (2 mm inner diameter) of the electrode. This prevented mechanical deformation (stripping) of the micro-thread of the piston mechanism during repeated extrusion of the dense carbon paste, which is an advantage over the rigid polymers traditionally used.

A pultruded carbon-fiber-reinforced plastic rod (2 mm in diameter) (Easy Composites Ltd., Stoke-on-Trent, UK), a composite of unidirectional carbon fibers in an epoxy-resin matrix, was used as the movable current collector. Replacing the traditional copper current-collecting wire with a carbon rod, which directly pushes the paste packed ahead of it inside the cavity, removes an oxidizable metal contact. The geometric area of the working surface (end face) was 3.14 mm^2^.

At the preliminary optimization stage (selection of the best modifier), the base paste was modified by adding aliquots of ethanolic solutions of DiPhytTMEDA, DiOctTMEDA, DiPerTMEDA, or tetrabutylammonium bromide (TBA), followed by thorough homogenization and thermal drying in an oven at 60 °C until the solvent was completely removed.

To prepare the DNA sensor, an aliquot of an ethanolic DiPhytTMEDA solution (equivalent to 0.048 mg, or 0.05 µmol, of dry modifier) and an aliquot of an aqueous solution of double-stranded salmon sperm DNA (equivalent to 0.15 mg, or 0.45 µmol on a phosphate-group basis) were successively introduced into 1 g of the base carbon paste. The target DNA sensor contained 0.05 µmol of DiPhytTMEDA per gram of paste, equivalent to 0.048 mg of dry modifier per gram. The mixture was thoroughly homogenized at room temperature—rather than oven-dried, in order to preserve the integrity of the incorporated DNA—until the solvents had visibly evaporated completely. This composition, selected empirically during optimization, provided the best balance between a high biopolymer packing density, retention of the electrical conductivity of the composite, and an optimal, stable analytical signal.

The resulting paste was tightly packed into the 3D-printed electrode body. Before each new measurement cycle, the working surface was renewed by micro-extruding a thin layer of paste with the screw mechanism, followed by mechanical polishing of the end face.

### 2.3. Electrochemical Measurements

All electrochemical measurements were performed with a CHI 660E potentiostat-galvanostat (CH Instruments Inc., Austin, TX, USA). Surface characterization and evaluation of the electrochemical properties of the CVPE were carried out by cyclic voltammetry (CV) and electrochemical impedance spectroscopy (EIS). Quantitative determination of the antitumor drugs was performed by differential pulse voltammetry (DPV).

For comparative evaluation of the effective surface area, a standard glassy carbon electrode (GCE, 3.14 mm^2^ geometric area, CH Instruments Inc., Austin, TX, USA) was used. Prior to measurements, the GCE working surface was carefully polished to a mirror finish with 0.05 µm alumina slurry on a polishing pad, thoroughly rinsed with deionized water, and ultrasonicated. All measurements were performed in a conventional three-electrode cell consisting of the CVPE (or the GCE, where indicated) as the working electrode, an Ag/AgCl (3 M KCl) reference electrode (CH Instruments Inc., Austin, TX, USA), and a platinum wire counter (auxiliary) electrode (CH Instruments Inc., Austin, TX, USA). Unless stated otherwise, all potentials are reported versus the [Ag/AgCl] reference electrode.

Estimation of the effective surface area and preliminary optimization of the composite composition were carried out in an equimolar mixture of 1 mM K_3_[Fe(CN)_6_] and 1 mM K_4_[Fe(CN)_6_] in a background of 0.1 M HEPES and 0.1 M KCl. Cyclic voltammograms were recorded over the potential range from −0.5 to 0.9 V at a scan rate of 100 mV/s. EIS measurements were performed in the presence of an equimolar mixture of 1 mM K_3_[Fe(CN)_6_] and 1 mM K_4_[Fe(CN)_6_] in a background of 0.1 M HEPES and 0.1 M KCl. Impedance spectra were recorded at the half-wave potential of the marker system over the frequency range from 100 kHz to 0.1 Hz with an AC amplitude of 5 mV.

### 2.4. Measurement of the Analytical Signal

To obtain the analytical signal (DPV), the CVPE filled with the DNA-modified paste was placed in an electrochemical cell containing 0.1 M HEPES buffer and 0.1 M KCl (pH 7.0) and the marker system—equimolar mixture of 1 mM K_3_[Fe(CN)_6_] and 1 mM K_4_[Fe(CN)_6_]. The anodic oxidation peak current of the [Fe(CN)_6_]^4−^ component was used as the analytical signal. A given analyte concentration was then added to the cell: doxorubicin over the range from 1 fM to 1 µM, or dacarbazine over the range from 1 nM to 1 µM. The linear calibration ranges were 1 pM–0.1 µM and 8 nM–1 µM, respectively. Measurements were carried out after preliminary incubation of the sensor in the analyte solution for 10 min under constant stirring to reach equilibrium of the intercalation and minor-groove binding processes. DPV was recorded with the following parameters: potential step 4 mV, amplitude 50 mV, pulse width 0.06 s, pulse period 0.5 s. For the optimization dependences, the absolute values of the anodic peak current (*I*_pa_), obtained after automatic subtraction of the capacitive background current in the CHI 660E software (CHI version 14.08), were used. For quantitative analysis, the current change was defined as Δ*I* = *I_i_* − *I*_0_, where *I*_0_ and *I_i_* are the marker peak currents before and after analyte incubation. Calibration plots were constructed using −Δ*I* for doxorubicin (signal-off) and Δ*I* for dacarbazine (signal-on). The sign of Δ*I* natively reflects the mechanism-dependent response (signal-off or signal-on), as detailed in Section 3.3. Using the relative quantity Δ*I* is necessary to mathematically normalize the response and to cancel out the microscopic fluctuations of the baseline signal *I*_0_ that inevitably arise during mechanical renewal of the paste-electrode surface before each new measurement. Calibration points are presented as mean ± SD (*n* = 7), and linear regression was performed over the stated concentration ranges.

Before recording the analytical signal by DPV, each mechanically renewed CVPE surface underwent a mandatory electrochemical stabilization procedure. The electrode was placed in the measuring cell with the background electrolyte (0.1 M HEPES, 0.1 M KCl, pH 7.0) containing an equimolar mixture of 1 mM K_3_[Fe(CN)_6_] and 1 mM K_4_[Fe(CN)_6_], and five successive cyclic-voltammetry scans were performed. The system reached a steady state of maximum electroactivity strictly on the fifth cycle, and the corresponding condition was used to record the stable baseline DPV current (*I*_0_) prior to the analyte addition.

### 2.5. Measurements in Synthetic Blood Plasma and Urine

The matrix effect in the determination of doxorubicin and dacarbazine was assessed by the standard-addition method using synthetic models of biological fluids. The artificial blood-plasma model contained NaCl (2 mM), NaHCO_3_ (0.2 mM), L-tryptophan (0.21 mM), L-tyrosine (0.2 mM), L-alanine (0.2 mM), L-aspartic acid (22 µM), DL-lysine (5 µM), L-arginine (5 µM), L-methionine (4 µM), L-phenylalanine (4 µM), L-histidine (3.5 µM), L-glycine (3.5 µM), and L-cysteine (1.3 µM) [33]. Artificial urine was prepared from KCl (21.5 mM), NaCl (50 mM), KH_2_PO_4_ (10.3 mM), CaCl_2_·2H_2_O (7.62 mM), NH_4_Cl (18.7 mM), and urea (416 mM) [34].

### 2.6. Scanning Electron Microscopy

Scanning electron microscopy (SEM) images of the electrode coatings were obtained with the high-resolution field emission scanning electron microscope Merlin^TM^ (Carl Zeiss, Oberkochen, Germany).

## 3. Results and Discussion

The DNA sensor developed here is a multicomponent system whose analytical properties are determined by the coordinated combination of the conductive carbon-veil matrix, the amphiphilic modifier, and the DNA receptor layer. Successive introduction of each component changes the accessibility of the electroactive surface, the charge-transfer conditions, and the interaction of the marker redox system with the electrode. Therefore, to establish the relationships governing the analytical response, the electrochemical characteristics of the composite had to be related to its composition and structural organization.

### 3.1. Electrochemical Behavior of the CVPE and Its Modification

**Figure 3:**
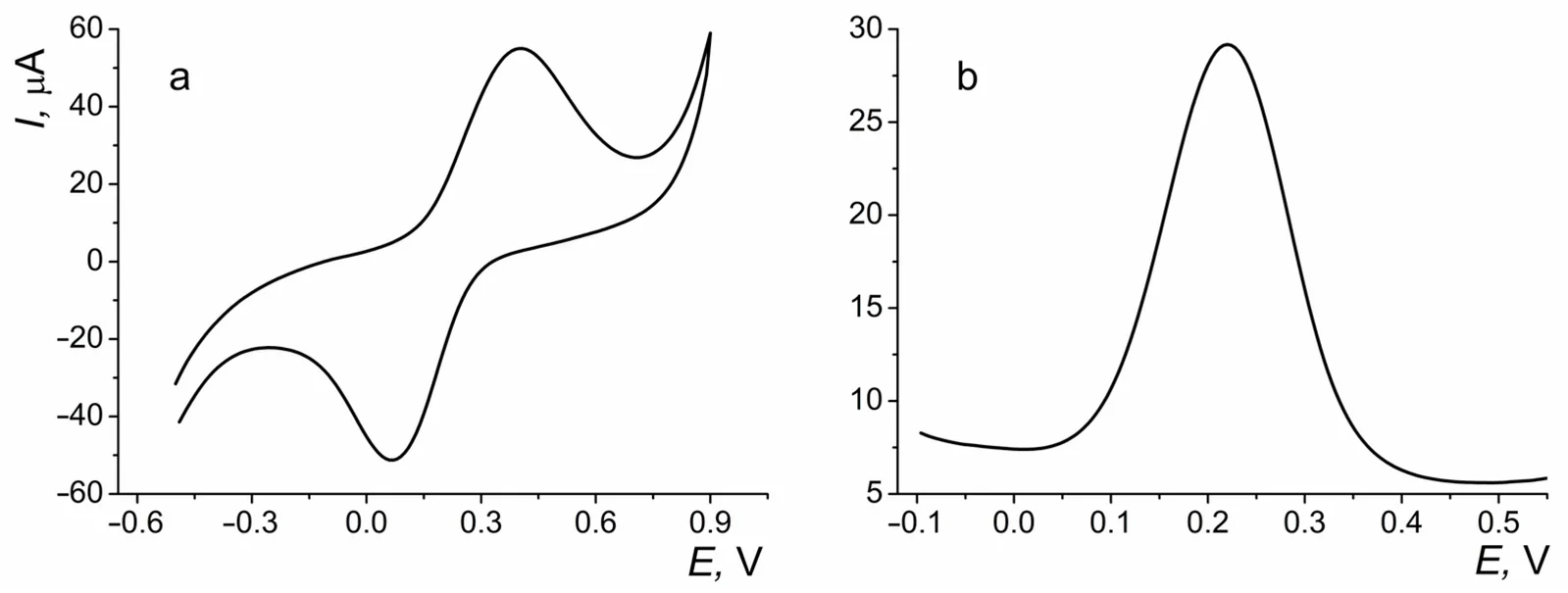
Electrochemical behavior of the unmodified CVPE (0.1 M HEPES, 0.1 M KCl, pH 7.0): (**a**) cyclic voltammogram in the presence of an equimolar mixture of 1 mM [Fe(CN)_6_]^3−/4−^; (**b**) differential pulse voltammogram in the presence of the equimolar mixture of 1 mM [Fe(CN)_6_]^3−/4−^.

The first stage in developing the sensor platform was to assess the electrochemical and physico-mechanical properties of the base carbon-veil composite. Because the ratio of conductive filler to binder simultaneously determines the charge-transfer efficiency, the accessibility of the pore space, and the stability of the paste during surface renewal, the liquid-paraffin content of the CVPE was optimized first. Preliminary cyclic voltammetry testing of various paraffin-to-carbon-veil mass ratios in the presence of the model [Fe(CN)_6_]^3−/4−^ redox system revealed that a 1:1 ratio provided the optimal balance between the electrical conductivity of the material and the mechanical stability of the paste. The typical electrochemical behavior of the unmodified CVPE prepared with this optimal 1:1 ratio is characterized by a well-defined voltammetric response, as illustrated in Figure 3.

Ground carbon veil was chosen as the conductive matrix in order to combine electrical conductivity, a developed surface, and mechanical stability of the bulk-modified paste. The carbon black (CB) traditionally used consists of near-spherical primary particles combined into aggregates and agglomerates, and the conductivity of composites based on it is governed by the formation of a percolation network and depends substantially on the particle distribution and the nature of the interparticle contacts [35,36]. Introducing an organic binder (such as liquid paraffin) and non-conductive components, including biopolymers, can increase the distance between conductive particles and reduce the connectivity of such a network. Unlike dispersed carbon powders, carbon veil is initially an interconnected network of microfibers with open interfiber gaps [20]. After grinding, the material retains an anisotropic, fibrous morphology rather than a near-spherical particulate one, which may favour the formation of a spatial contact network throughout the paste. The interfiber gaps can provide electrolyte access to the internal regions of the composite, and the fibrous structure can help mechanically stabilize the plastic paraffin matrix during its repeated renewal.

Incorporating the highly porous carbon veil into the paste led to a substantial increase in the active electrode surface. The effective working-surface area was calculated from the Randles-Ševčík equation (*I*_p_ = (2.69 × 10^5^) × n^3/2^ × *A* × *D*^1/2^ × *c* × *v*^1/2^), where *I*_p_ is the peak current (A); 2.69 × 10^5^ is the constant for 25 °C; *n* = 1 is the number of transferred electrons; *A* is the effective electrode surface area (cm^2^); *D* = 7.6 × 10^−6^ is the diffusion coefficient (cm^2^/s); *c* is the concentration of the substance in solution (mol/cm^3^); and *v* is the potential scan rate (V/s). The calculated effective electrochemical area of the developed system exceeded its nominal geometric area more than fivefold (Table 1). It should be noted that the Randles–Ševčík equation assumes semi-infinite planar diffusion; in the fibrous, porous carbon-veil matrix, thin-layer-like behaviour in the interfiber voids may lead to an overestimation of the apparent electroactive surface area.

For comparison, for a glassy carbon electrode (GCE) with a dense, low-porosity structure, this ratio is traditionally below unity (about 0.94). This clearly demonstrates the advantage of using three-dimensional carbon materials in electroanalysis: a several-fold increase in the electroactive area enlarges the electrochemically accessible interface and increases the recorded current density, creating the prerequisites for an enhanced analytical signal [37].

**Figure 4:**
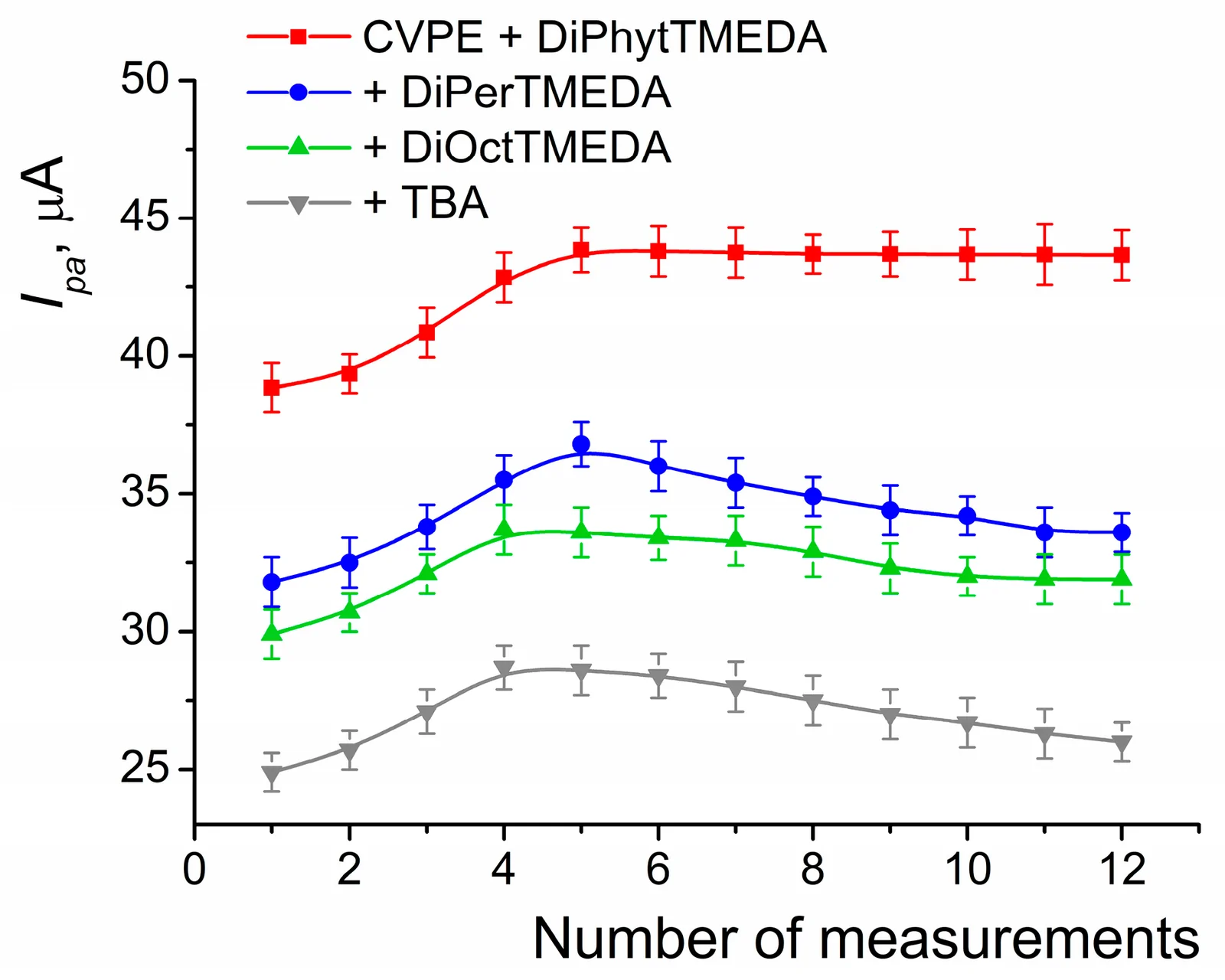
Dependence of the anodic peak current (*I*_pa_) of the equimolar mixture of 1 mM [Fe(CN)_6_]^3−/4−^ on the number of successive measurement cycles for the CVPE modified with different compounds (0.1 M HEPES, 0.1 M KCl, pH 7.0). Error bars represent the standard deviation (SD, *n* = 5).

In the next stage, the influence of the nature of the amphiphilic modifiers on the electrochemical response of the CVPE was evaluated. To justify the chosen measurement protocol and to assess the operational stability of the composite, the dependence of the anodic peak current on the number of successive scan cycles was studied (Figure 4). Measurements were carried out continuously in the same [Fe(CN)_6_]^4−/3−^ marker solution without mechanical renewal of the electrode surface.

For all modifier types, the analytical signal increased sharply during the first cycles, reaching a pronounced maximum on the fifth scan. This increase is due to electrochemical activation of the composite: gradual penetration of the aqueous electrolyte into the paste micropores, removal of excess dielectric paraffin binder from the interface, and conformational reorientation of the amphiphile molecules.

After reaching this initial maximum of activation, the responses of the electrodes modified with DiOctTMEDA, DiPerTMEDA, and TBA began to decline steadily. This degradation is associated with gradual leaching of the less lipophilic components from the near-surface layer of the matrix into the bulk solution. In contrast, the DiPhytTMEDA modifier, owing to its extended, branched phytyl radical, is firmly retained in the bulk of the composite. After the activation stage, the signal of the DiPhytTMEDA-modified electrode established a true steady state and remained virtually unchanged in subsequent cycles, which was the basis for selecting this modifier for the further stages of the work.

**Figure 5:**
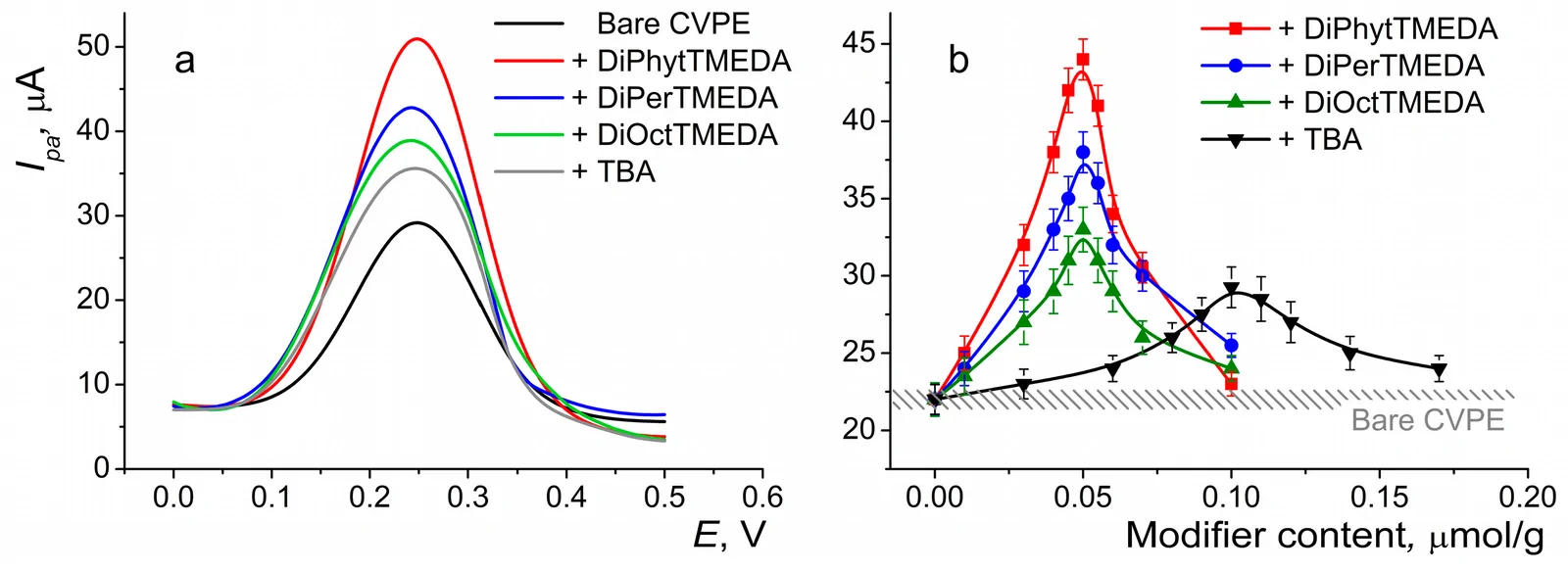
Influence of the nature and concentration of the amphiphilic modifiers on the electrochemical response of the CVPE (0.1 M HEPES, 0.1 M KCl, equimolar mixture of 1 mM [Fe(CN)_6_]^3−/4−^, pH 7.0): (**a**) differential pulse voltammograms on the unmodified electrode and on electrodes modified with different surfactants at the amounts corresponding to the maximum analytical signal; (**b**) dependences of the anodic peak current (*I*_pa_, after background baseline subtraction) on the modifier content in the bulk of the paste. The hatched area represents the blank signal of the electrode without modifier (mean ± SD). Error bars represent the standard deviation (SD, *n* = 5).

Taking into account the established protocol of mandatory electrochemical surface activation, concentration dependences were obtained for all modifiers studied (Figure 5). The analytical signal was the anodic peak current (*I*_pa_) recorded strictly on the fifth scan cycle, corresponding to the steady state of maximum electroactivity. As the voltammograms show (Figure 5a), introducing the dicationic surfactants into the paste substantially increased the marker oxidation peak currents compared with the unmodified CVPE. The best electrochemical characteristics and the maximum analytical signal were provided by the phytol-based amphiphilic derivative (DiPhytTMEDA). The plots of current versus modifier content (per 1 g of paste) are distinctly extremal (Figure 5b). For all the amphiphilic TMEDA derivatives studied, the maximum analytical response was reached at a concentration of 0.05 µmol/g, whereas for TBA the optimum is shifted to twice that concentration.

The observed differences are consistent with the molecular structure and the interfacial interaction mechanism of the modifiers. Being a monocation, TBA exhibits no pronounced surface-active properties in the hydrophobic matrix. In contrast, the dicationic amphiphilic derivatives, owing to their two charged centers and extended lipophilic radicals, can orient effectively at the paste/solution interface. Such a layer architecture promotes local electrostatic concentration of the marker anions and improved electron transfer at a substantially lower modifier concentration. A further increase in the surfactant fraction in the paste leads to the expected decrease in peak currents due to blocking of the electroactive zones by the excess non-conductive organic phase.

Among the compounds studied, DiPhytTMEDA provided the highest currents and the most stable results in a series of successive measurements (Figure 5), which was the basis for its use in the subsequent stages of DNA-sensor construction.

### 3.2. Effect of Introducing DNA into the Modified Paste and Analysis of the Composite Morphology

In the next stage, the content of DNA in the bulk-modified paste was optimized. Double-stranded salmon sperm DNA was chosen because of the mechanisms by which the studied drugs interact with DNA: doxorubicin intercalates between the base pairs of the double helix [38], whereas dacarbazine can form complexes in the DNA minor groove involving hydrogen bonds and van der Waals interactions [39]. The interaction of dacarbazine with double-stranded salmon sperm DNA has also previously been recorded by voltammetric and spectroscopic methods [40]. Salmon sperm DNA, in turn, has a relatively short chain length (on average about 2000 base pairs) compared with genomic DNA. A lower DNA molecular mass is accompanied by a decrease in its intrinsic viscosity and in the extent of interchain interactions [41], so the use of a fragmented biopolymer facilitates its homogeneous introduction into the paste matrix.

**Figure 6:**
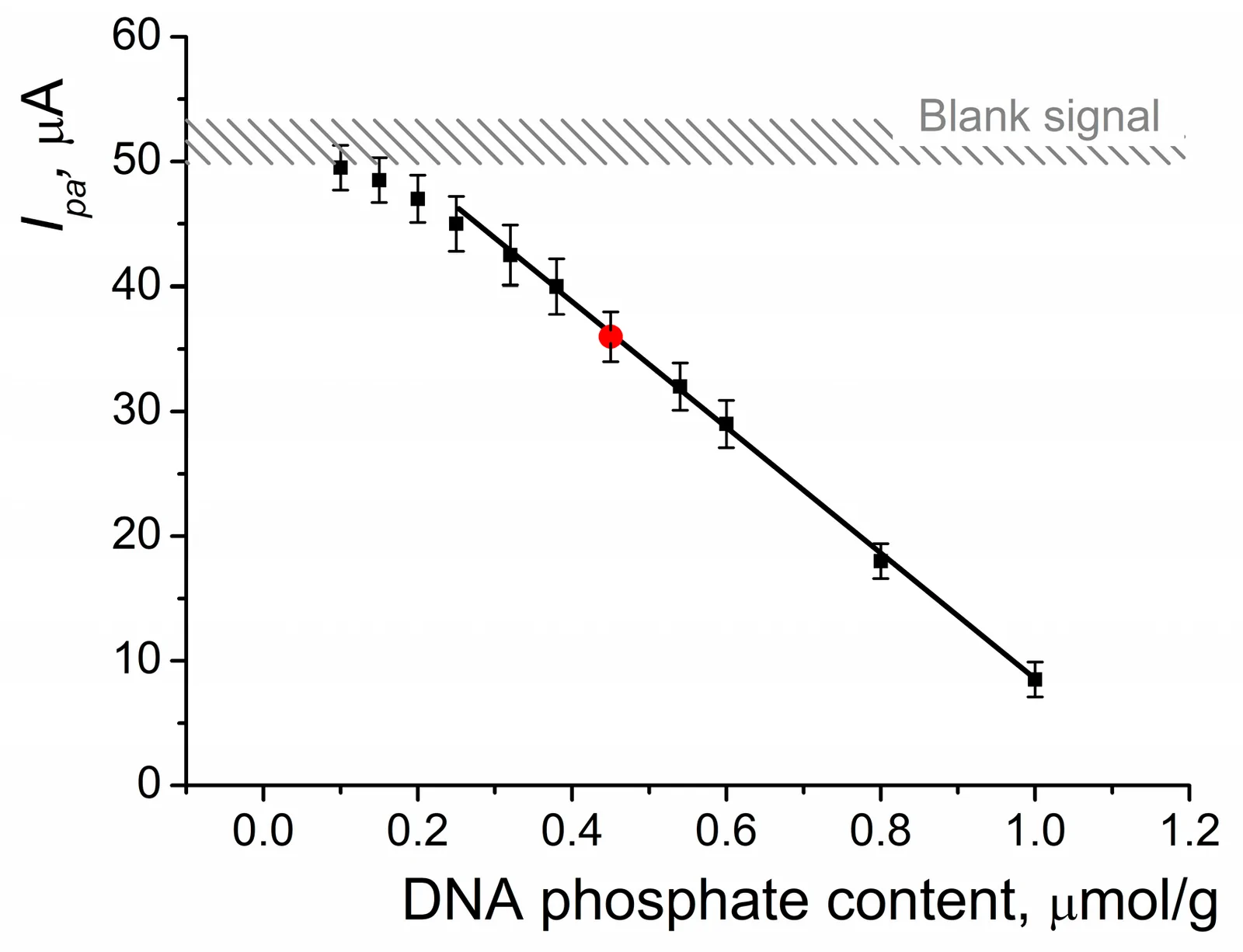
Influence of the native-DNA content in the bulk of the composite (on a phosphate-group basis) on the anodic oxidation peak current (*I*_pa_) of 1 mM [Fe(CN)_6_]^4−^ for the CVPE pre-modified with DiPhytTMEDA (0.1 M HEPES, 0.1 M KCl, pH 7.0). The red marker highlights the biopolymer concentration (0.45 µmol/g) chosen for further construction of the receptor layer. The solid black line guides the eye through the decreasing-current region. The hatched area represents the blank signal of the electrode without DNA (mean ± SD). Error bars represent the standard deviation (SD, *n* = 5).

As the dependence of the [Fe(CN)_6_]^4−^ anodic oxidation peak current on the concentration of the phosphate groups of the biopolymer in the bulk of the paste shows (Figure 6), introducing DNA at up to 0.1 µmol/g causes virtually no screening of the electrode surface. However, with a further increase in the receptor-component content, the marker oxidation peak currents decrease linearly.

To ensure a high receptor-layer density and a sufficient excess of biopolymer in the bulk of the composite, a DNA phosphate-group concentration of 0.45 µmol/g was selected for further work. This point lies within the linear descending region of the screening curve (Figure 6), which guarantees high reproducibility of the receptor layer formation while maintaining sufficiently high marker-system currents (above 35 µA).

Based on the optimization data obtained, a composite paste based on ground carbon veil was prepared with the amphiphilic modifier DiPhytTMEDA (0.05 µmol/g) and DNA (0.45 µmol/g on a phosphate-group basis) introduced jointly directly into the bulk of the material. The selected concentrations of all introduced components provide the best ratio between the receptor-coating density, the magnitude of the recorded analytical signal, and the stability of the system under the selected analytical conditions.

**Figure 7:**
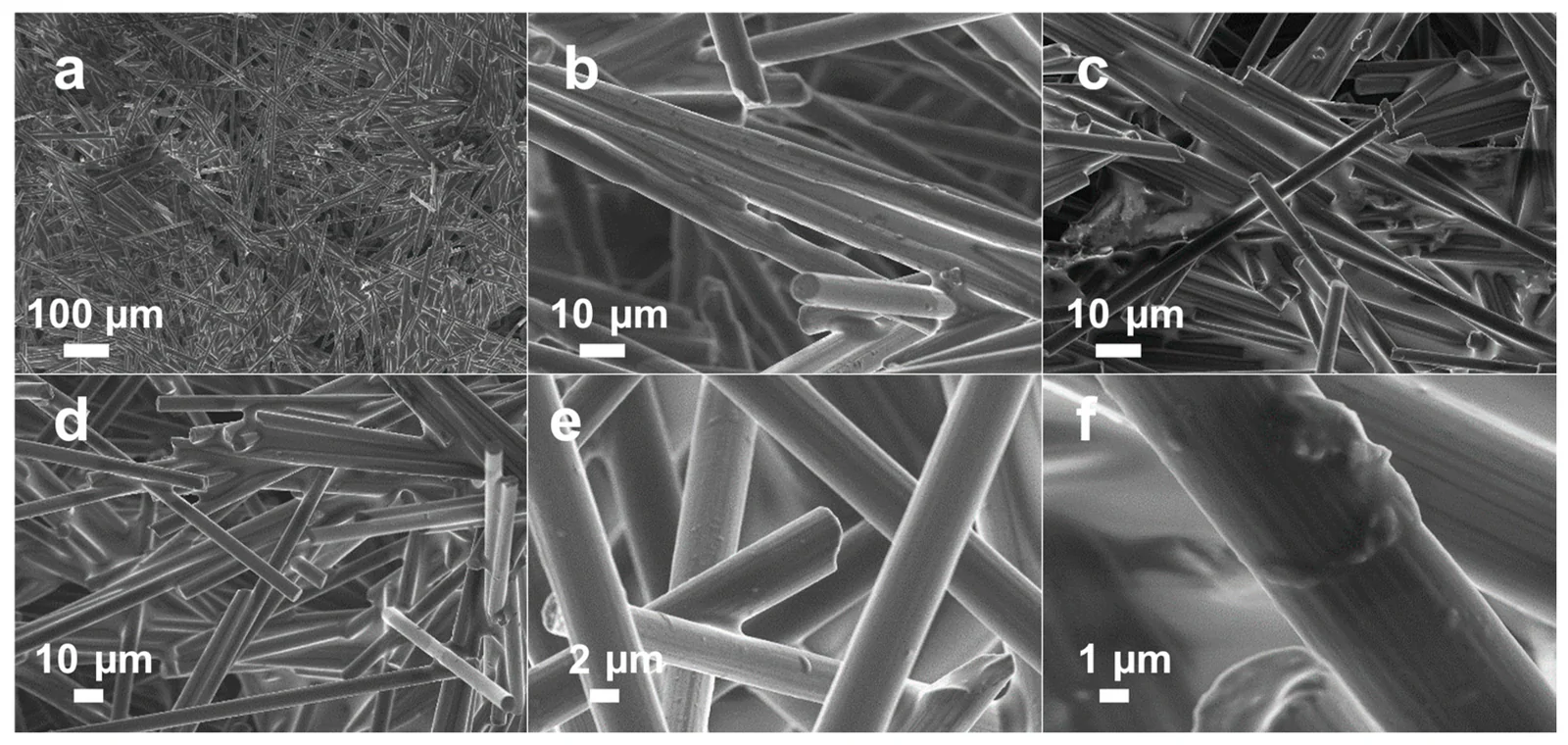
SEM micrographs of the electrode-surface morphology: (**a**,**d**) unmodified base carbon paste; (**b**,**e**) paste modified with the amphiphile DiPhytTMEDA; (**c**,**f**) the target bulk-modified DNA sensor. The top row illustrates preservation of the macroporous structure (low magnification); the bottom row shows changes in the microrelief of the fiber surface (high magnification).

Formation of the receptor layer at the successive modification stages of the composite was confirmed by SEM (Figure 7).

On the pristine, unmodified CVPE (Figure 7a,d), the matrix is a highly porous three-dimensional scaffold formed by randomly interwoven cylindrical fragments of carbon microfibers. Liquid paraffin acts solely as a structural binder at the nodes of their intersection. This open fibrous architecture accounts for the substantial increase in electroactive area calculated earlier (Table 1).

Introducing the lipophilic amphiphile DiPhytTMEDA (Figure 7b,e) preserves the original macroporosity without clogging the channels for marker diffusion. However, at high magnification, the formation of characteristic island-like globular structures of the modifier is visualized on the smooth surface of the carbon fibers. A further confirmation of successful formation of the organic coating is the noticeable increase in the brightness of the SEM images (increased secondary-electron yield), characteristic of the local charging effect of dielectric lipophilic layers under the electron probe.

Finally, when the DNA is incorporated into the composite (Figure 7c,f), the formation of massive amorphous films and agglomerates is observed on the surface of the conductive carbon rods. This morphology reflects successful immobilization of the biopolymer: the polyanionic DNA chains bind, through strong electrostatic interactions, to the dicationic centers of DiPhytTMEDA, forming a stable receptor coating directly on the electroactive elements of the bulk matrix.

### 3.3. Determination of Doxorubicin and Dacarbazine

To assess the analytical capabilities of the developed bulk-modified CVPE, its voltammetric characteristics were studied in the presence of different concentrations of the antitumor drugs. The analytical signal was recorded by DPV from the change in the oxidation peak current of the [Fe(CN)_6_]^4−^ marker system (1 mM).

It should be emphasized that while doxorubicin and dacarbazine are intrinsically electroactive compounds, their direct anodic currents at the ultra-low working concentrations (from picomolar up to 1 µM) are entirely negligible against the massive voltammetric response of the 1 mM [Fe(CN)_6_]^4−^ marker. Thus, the recorded signal strictly reflects the modulation of the marker’s diffusion and charge transfer, without any intrinsic faradaic interference from the drugs.

**Figure 8:**
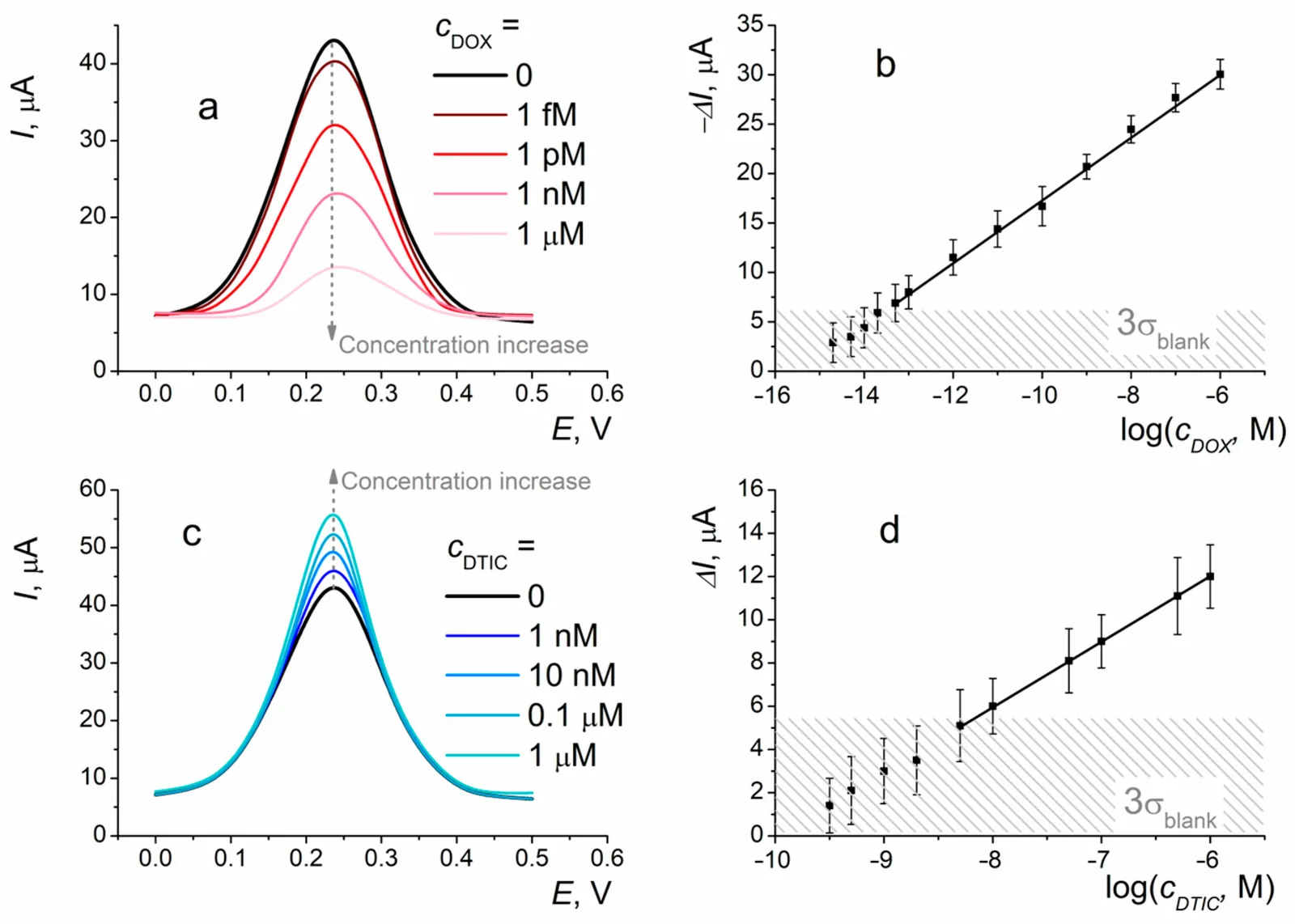
Electrochemical determination of the antitumor drugs on the bulk-modified DNA sensor (CVPE + DiPhytTMEDA + DNA; 0.1 M HEPES, 0.1 M KCl, pH 7.0): differential pulse voltammograms of the equimolar mixture of 1 mM [Fe(CN)_6_]^3−/4−^ marker system upon addition of increasing concentrations of (**a**) doxorubicin and (**c**) dacarbazine; the corresponding calibration plots of the change in anodic peak current (Δ*I*, expressed as −Δ*I* for (**b**) and Δ*I* for (**d**)) versus the logarithm of concentration for (**b**) doxorubicin and (**d**) dacarbazine. The solid black lines in (**b**,**d**) are linear regression fits. The hatched areas represent the 3σblank regions. Error bars represent the standard deviation (SD, *n* = 7).

As the doxorubicin content in solution increased, the marker-anion oxidation peak currents decreased linearly (Figure 8a). This effect is due to the pronounced intercalating ability of the anthracycline antibiotic. Doxorubicin molecules efficiently insert between the base pairs of the DNA double helix distributed in the bulk and on the surface of the composite, causing the polymer chain to thicken and stiffen. Upon intercalation, these steric hindrances partially block the initially open pore channels of the CVPE, impeding diffusion of the marker anions.

The calibration plots (Figure 8b,d) were constructed by plotting the change in the anodic peak current against the logarithm of the drug concentration. To accurately reflect the mechanism-dependent, oppositely directed responses without losing the sign of the signal, the difference was calculated as −Δ*I* = *I*_0_ − *I_i_* for doxorubicin (Signal-OFF) and Δ*I* = *I*_0_ − *I_i_* for dacarbazine (Signal-ON). The corresponding linear regression equations were:

−Δ*I* (µA) = (3.21 ± 0.03) × log(*c*(DOX)) + (49.81 ± 0.29) (R^2^ = 0.998) (1)

Δ*I* (µA) = (3.01 ± 0.01) × log(*c*(DTIC)) + (30.08 ± 0.07) (R^2^ = 0.998) (2)

The limits of detection (LOD), calculated from the intersection of the linear calibration fits with the 3*σ*_blank_ threshold (5.7 µA, evaluated from 7 independent measurements of the background signal), were 19 fM for doxorubicin and 8.0 nM for dacarbazine. The corresponding linear ranges started at 1 pM and 8 nM, respectively.

The low calculated LOD for doxorubicin may arise from amplification of the electrochemical response associated with cooperative changes in the DNA-containing layer. The opposite DPV responses of the two drugs are consistent with their different effects on the DNA-containing layer. Doxorubicin intercalation can increase local DNA packing and restrict access of the [Fe(CN)_6_]^4−^ marker to the conductive carbon matrix, producing a signal-off response. In contrast, dacarbazine binding in the minor groove may alter the local charge distribution and facilitate marker transport, producing a signal-on response.

The background electrolyte with increased buffer capacity and ionic strength (0.1 M HEPES, 0.1 M KCl), compared with the dilute 0.01 M solutions traditionally used in the literature, was chosen deliberately. Creating a massive excess of background components provides competitive passivation of the surface of laboratory plastic (tubes and pipette tips). This makes it possible to critically minimize losses of the target drugs due to their nonspecific adsorption on the vessel walls, which is a necessary condition for correctly maintaining the concentration gradient when working in the ultralow (femto- and nanomolar) analyte-concentration ranges [42]. The choice of the working value of pH 7.0, in turn, is due to the need to bring the analysis conditions as close as possible to the near-neutral conditions relevant to the analysis of biological samples [43]. In addition, maintaining a neutral medium satisfies the critical requirements for preserving the native conformation of double-stranded DNA, reducing the likelihood of phosphate-backbone degradation and unwinding of the hydrogen bonds of the biopolymer during the measurements [44].

**Figure 9:**
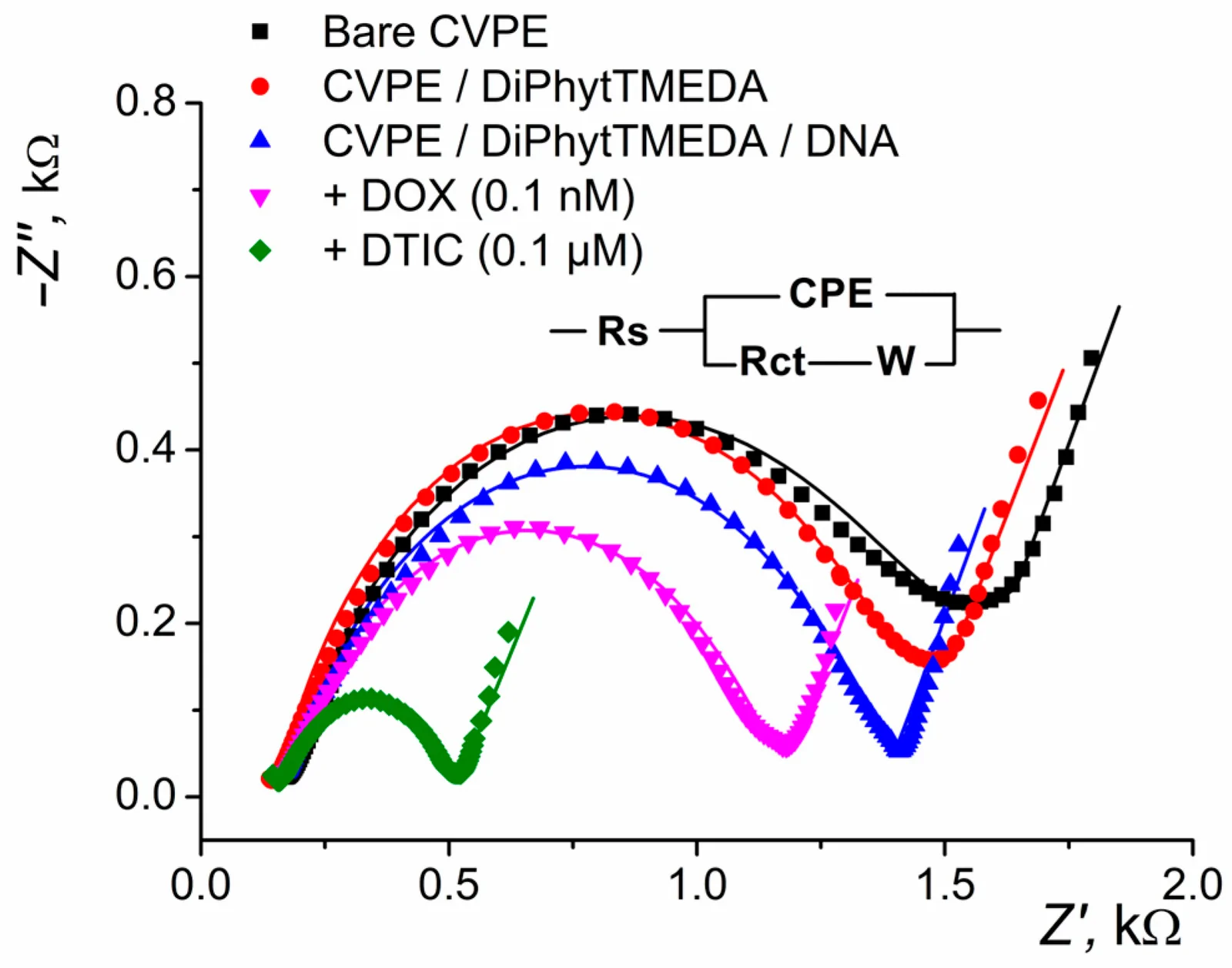
Nyquist plots for the unmodified CVPE, the CVPE + DiPhytTMEDA, and the target DNA sensor before and after a 10-min incubation upon the addition of 0.1 nM doxorubicin and 0.1 µM dacarbazine into the measuring cell (0.1 M HEPES, 0.1 M KCl, an equimolar mixture of 1 mM K_3_[Fe(CN)_6_] and 1 mM K_4_[Fe(CN)_6_], pH 7.0). Symbols denote experimental data; solid lines are the results of theoretical fitting. The inset shows the equivalent electrical circuit used.

Electrochemical impedance spectroscopy was used to further examine drug-induced changes at the electrode interface (Figure 9, Table 2). To clearly visualize the specific effect of the drugs on the charge-transfer resistance, impedance spectra were recorded at analyte concentrations (0.1 nM for doxorubicin and 0.1 µM for dacarbazine) providing a pronounced response within their respective linear ranges. Analysis of the Nyquist plots showed that the interaction of the biosensor with the target analytes leads to a specific change in the charge-transfer resistance (*R*_ct_), the character of which depends strictly on the nature of the drug.

The difference in the magnitude of the *R*_ct_ decrease for dacarbazine (to 0.36 kΩ) and doxorubicin (to 0.98 kΩ) should be considered in view of the different test concentrations (0.1 µM and 0.1 nM, respectively), selected within their respective response ranges. At the tested concentration, dacarbazine binding may alter the organization of the DNA-containing layer and increase the accessibility of the electroactive carbon matrix. For doxorubicin, the moderate decrease in *R*_ct_ may reflect the combined influence of restricted marker transport and local electrostatic interactions. Doxorubicin intercalation can increase DNA rigidity and hinder marker diffusion through the porous layer, consistent with the current decrease observed by DPV. At the same time, the protonated daunosamine group provides localized positive charge, which may facilitate the approach of [Fe(CN)_6_]^4−^ to the interface and contribute to the decrease in *R*_ct_. These results illustrate the complementary information provided by DPV and EIS on marker transport through the receptor layer and interfacial charge transfer.

**Figure 10:**
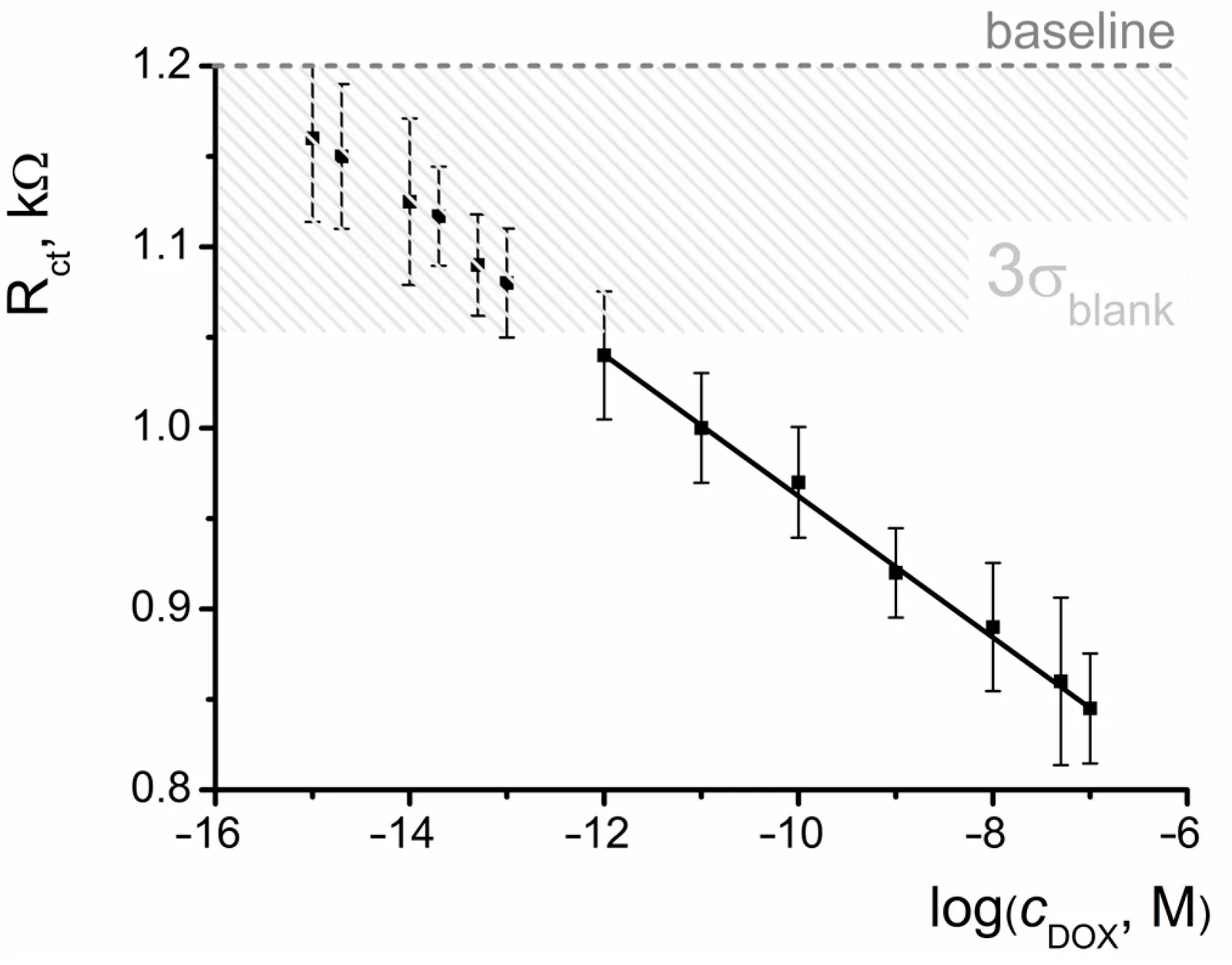
Dependence of the charge-transfer resistance (*R*_ct_) on the logarithm of the doxorubicin concentration for the bulk-modified DNA sensor (0.1 M HEPES, 0.1 M KCl, an equimolar mixture of 1 mM K_3_[Fe(CN)_6_] and 1 mM K_4_[Fe(CN)_6_], pH 7.0). The solid black line is the linear regression fit. The horizontal dashed line denotes the baseline signal recorded before analyte addition, whereas the hatched area represents the 3σblank region. Error bars represent the standard deviation (SD, *n* = 7).

To further characterize the impedimetric response, the dependence of *R*_ct_ on the doxorubicin concentration was examined (Figure 10). As the plot shows, the drop in charge-transfer resistance depends linearly on the logarithm of the drug concentration. The corresponding linear regression equation was:

*R*_ct_ (kΩ) = −(0.039 ± 0.001) × log(*c*(DOX)) + (0.57 ± 0.01), (R^2^ = 0.998) (3)

The impedimetric LOD was determined as the intersection of the 3σblank level with the line defined by (3) (1.08 kΩ, corresponding to the baseline *R*_ct_ of 1.20 kΩ minus three standard deviations of 0.04 kΩ).

The calculated EIS LOD was 115 fM. This value is substantially higher than the LOD achieved by DPV (19 fM). This quantitative difference confirms our initial assumption and substantiates the use of electrochemical impedance spectroscopy primarily for interfacial mechanistic studies and validation of binding models, whereas differential pulse voltammetry remains the method of choice for ultrasensitive quantification. The stable dose-dependent trend in EIS (R^2^ = 0.998) suggests that, under equilibrium impedance conditions, electrostatic attraction of the marker anions by protonated doxorubicin predominates over the steric pore-blocking contribution.

Because the interaction of doxorubicin and dacarbazine with the DNA matrix causes oppositely directed changes in the marker diffusion current (a decrease and an increase, respectively), their simultaneous presence in the analyzed solution produces a superposition of the analytical signals. For this reason, direct voltammetric separation of the doxorubicin and dacarbazine responses in complex mixtures (for example, in the biological fluids of patients undergoing combined polychemotherapy under the ABVD protocol [45]) without prior chromatographic separation is difficult. Other DNA-binding compounds may also contribute to the response of the DNA-containing sensing layer, which should be considered when analysing samples of complex composition. The use of combination regimens is generally regarded as one of the factors complicating the therapeutic drug monitoring of cytostatics [46]. Accordingly, the developed platform is most applicable to the determination of one of the studied drugs in the absence of the other, or after preliminary separation of the mixture components. Its potential fields of application are the monitoring of single-agent therapy and the rapid analysis of pharmaceutical substances.

It should be emphasized that the choice of doxorubicin and dacarbazine in this study is primarily of a fundamental, model nature. These drugs were used as classic representatives of two fundamentally different mechanisms of interaction with the bioreceptor: doxorubicin acts as a rigid intercalator, whereas dacarbazine models non-covalent minor-groove binding to the biopolymer. Under physiological in vivo conditions, dacarbazine is a prodrug, and its direct cytotoxic alkylating activity is realized only after microsomal oxidation in the liver to form the active metabolite. Nevertheless, as physico-chemical in vitro studies show [39], the parent, unmetabolized dacarbazine molecule also has a pronounced affinity for DNA, forming stable complexes through hydrophobic interactions and hydrogen bonds along the phosphate backbone. The ability of the proposed platform to distinguish such subtle differences between the conformational changes in DNA upon intercalation and upon non-covalent groove binding highlights its versatility and its potential for targeted pharmacokinetic screening.

### 3.4. Analytical Application in Synthetic Model Matrices and Comparison with Analogues

To evaluate the tolerance of the biosensor to potential matrix interferents, doxorubicin and dacarbazine were determined in synthetic blood plasma (Table 3) and synthetic urine (Table 4) using the standard addition method. It should be noted that the artificial plasma model used primarily reflects the inorganic and amino acid composition of the fluid but lacks macromolecular proteins such as albumin. Since doxorubicin is known to be highly protein-bound in true clinical samples, these measurements serve as a proof-of-concept for the sensor’s selectivity against small-molecule interferents. To evaluate the matrix concentration effect, a fixed amount of the analyte (10.0 nM) was introduced into matrix solutions diluted 1:10 and 1:100.

Recoveries of doxorubicin and dacarbazine in the synthetic plasma and urine models were 94–99%, indicating that the tested matrix components had no marked effect on the analytical response under the selected conditions. The fabricated DNA sensor based on the CVPE and the DiPhytTMEDA modifier is suitable for the high-accuracy analysis of drugs in samples of complex biological fluids without laborious preliminary sample-preparation steps.

Table 5 compares the analytical characteristics of the developed DNA sensor with those of other electrochemical sensors known from the literature for the determination of doxorubicin and dacarbazine.

Among the methods compared in Table 5, the developed sensor provided the lowest detection limit for doxorubicin (19 fM). For dacarbazine, the detection limit of 8.0 nM was lower than the 35 nM value reported for one CNT-based system [51] but higher than the 0.09 and 0.12 nM values reported for the other nanostructured electrodes [50,52].

An important operational characteristic of the developed bulk-modified DNA sensor is the interfacial stability of the organic components. As shown earlier (Figure 4), continuous cyclic scanning of a single unrenewed surface exhibits high stability of the analytical signal. In a series of ten successive measurements after initial activation, the relative standard deviation (RSD) of the analytical response does not exceed 3–5%, confirming the firm retention of the bioreceptor layer and the DiPhytTMEDA modifier in the hydrophobic bulk of the paste, as well as the absence of component leaching into the buffer solution. Between-electrode reproducibility was evaluated using five independently prepared sensors at a doxorubicin concentration of 0.1 nM. The RSD of ΔI was 5.7%. The long-term stability (shelf life) of the sensor when stored under refrigerator conditions (4 °C) was estimated to be at least one month, with retention of more than 90% of the initial sensitivity, and was limited solely by the natural structural-degradation processes of the DNA.

An important technological advantage of the developed platform is the use of a custom 3D-printed electrode body equipped with an internal screw-threaded piston mechanism. This design feature allows precise micro-extrusion of the composite paste. Unlike traditional carbon paste electrode designs, in which mechanical renewal of the working surface inevitably involves significant and uncontrolled material consumption, the fine thread pitch of the 3D-printed body makes it possible to extrude a strictly metered, microscopic layer of spent paste. Subsequent mechanical polishing of the end face instantly exposes a fresh, reproducible receptor surface. This approach is designed to ensure maximum economy of the expensive biocomponents, potentially reducing the cost of a single analysis severalfold and allowing multiple independent measurements to be performed with a single filled biosensor, which is a critically important factor for introducing the development into routine laboratory practice.

## 4. Conclusions

In this work, a new, highly efficient electroanalytical platform was developed and successfully tested for the determination of antitumor drugs (doxorubicin and dacarbazine) whose mechanism of action is based on specific binding to the DNA double helix. The developed system is a comprehensive engineering-chemical solution that compares favorably with traditional analogues based on glassy carbon and graphite pastes.

The sensor design, implemented by FDM 3D printing from chemically inert thermoplastic polyurethane with a carbon current collector, reduced the risk of background anodic currents from oxidizable metal parts. The built-in screw-piston mechanism enabled precise micro-extrusion of the composite paste, allowing the receptor layer to be renewed repeatedly and reproducibly with minimal consumption of the expensive biocomponents.

The use of ground carbon veil as the conductive base ensured the formation of an open, highly porous three-dimensional composite architecture, resulting in a fivefold increase in the effective electroactive area. Subsequent bulk modification of the paste with the original dicationic amphiphile DiPhytTMEDA and the DNA made it possible to create a stable polyelectrolyte receptor complex that retains high permeability to the [Fe(CN)_6_]^4−^ marker ions.

Mechanism-dependent steric and electrostatic modulation of the marker response yielded DPV detection limits of 19 fM for doxorubicin and 8.0 nM for dacarbazine. The femtomolar doxorubicin response extends the analytical window toward trace-level measurements, whereas dacarbazine was determined in the nanomolar range. Furthermore, testing in 10- and 100-fold diluted synthetic plasma and urine demonstrated excellent recoveries (94–99%), confirming the system’s tolerance to the inorganic and amino acid components of synthetic matrices.

The developed sensor combines high sensitivity with the ability to record differences in the interaction of the antitumor drugs with the DNA-containing layer. Given the possible compensation of the oppositely directed responses when doxorubicin and dacarbazine are present together, its most promising applications are the rapid quality control of pharmaceutical substances, and the pharmacokinetic screening of monotherapy.

## Figures and Tables

**Table 1:** Comparison of the geometric (*A*) and effective (*A*_eff_) working-surface areas of the GCE and the CVPE.

Electrode	*A*, cm^2^	*A*_eff_, cm^2^	*A*_eff_/*A*
GCE	0.031	0.029	0.94
CVPE	0.031	0.157	5.06

**Table 2:** Electrochemical charge-transfer parameters at the electrode/solution interface. CPE-constant phase element; *R*_s_-solution resistance; *R*_ct_-charge-transfer resistance. Error values represent the standard deviation (SD, *n* = 7).

Electrode	CPE, µF	*R*_s_, Ω	*R*_ct_, kΩ
CVPE	3.0 ± 0.2	164 ± 4	1.35 ± 0.03
CVPE + DiPhytTMEDA	3.0 ± 0.2	159 ± 3	1.29 ± 0.04
CVPE + DiPhytTMEDA + DNA	1.3 ± 0.1	164 ± 5	1.20 ± 0.04
CVPE + DiPhytTMEDA + DNA in doxorubicin solution (0.1 nM)	1.5 ± 0.1	162 ± 5	0.98 ± 0.05
CVPE + DiPhytTMEDA + DNA in dacarbazine solution (0.1 µM)	3.3 ± 0.3	155 ± 5	0.36 ± 0.03

**Table 3:** Accuracy of the determination of doxorubicin and dacarbazine in synthetic blood plasma with the developed biosensor system. Error values represent the standard deviation (SD, *n* = 7).

Analyte	Dilution	Added, nM	Found, nM	Recovery, %
Doxorubicin	1:10	10.0	9.6 ± 0.2	96 ± 2
Doxorubicin	1:100	10.0	9.9 ± 0.1	99 ± 1
Dacarbazine	1:10	10.0	9.4 ± 0.3	94 ± 3
Dacarbazine	1:100	10.0	9.8 ± 0.2	98 ± 2

**Table 4:** Accuracy of the determination of doxorubicin and dacarbazine in synthetic urine with the developed biosensor system. Error values represent the standard deviation (SD, *n* = 7).

Analyte	Dilution	Added, nM	Found, nM	Recovery, %
Doxorubicin	1:10	10.0	9.7 ± 0.2	97 ± 2
Doxorubicin	1:100	10.0	9.8 ± 0.2	98 ± 2
Dacarbazine	1:10	10.0	9.6 ± 0.3	96 ± 3
Dacarbazine	1:100	10.0	9.7 ± 0.2	97 ± 2

**Table 5:** Comparison of the analytical characteristics of doxorubicin and dacarbazine determination using the developed sensor with literature data.

Analyte	Coating Composition	Linear Range	*c*_lim_	Ref.
Doxorubicin	Carbon black, chitosan, aminated thiacalix[4]arene	10 pM–1 nM	0.3 pM	[47]
Doxorubicin	Single-walled carbon nanotubes	1.0 nM–20 µM	0.6 nM	[48]
Doxorubicin	Polyaniline	1.0 pM–1 mM	0.6 pM	[49]
Doxorubicin	CVPE + DiPhytTMEDA + DNA	1 pM–0.1 µM	19 fM	This work
Dacarbazine	Graphene, gold, PEDOT	2.5 nM–1.5 µM	0.09 nM	[50]
Dacarbazine	CNT paste, poly(2-amino-1,3,4-thiadiazole)	0.05–24 µM	35 nM	[51]
Dacarbazine	Carbon paste electrode, CNT	0.4 nM–2.5 µM	0.12 nM	[52]
Dacarbazine	CVPE + DiPhytTMEDA + DNA	8 nM–1 µM	8.0 nM	This work

## Data Availability

Dataset available upon request from the authors.

## Abbreviations

The following abbreviations are used in this manuscript:

ABVD	doxorubicin (Adriamycin), bleomycin, vinblastine, and dacarbazine chemotherapy regimen
AC	alternating current
CB	carbon black
CNT	carbon nanotube
CPE	constant phase element
CV	cyclic voltammetry
CVPE	carbon veil paste electrode
DiOctTMEDA	octyl-derived dicationic amphiphilic modifier
DiPerTMEDA	perillyl-derived dicationic amphiphilic modifier
DiPhytTMEDA	phytol-derived dicationic amphiphilic modifier
DNA	deoxyribonucleic acid
DPV	differential pulse voltammetry
EIS	electrochemical impedance spectroscopy
FDM	fused deposition modeling
GCE	glassy carbon electrode
HEPES	4-(2-hydroxyethyl)-1-piperazineethanesulfonic acid
HPLC	high-performance liquid chromatography
LC-MS	liquid chromatography–mass spectrometry
PEDOT	poly(3,4-ethylenedioxythiophene)
RSD	relative standard deviation
SEM	scanning electron microscopy
TBA	tetrabutylammonium bromide
TMEDA	*N*,*N*,*N*′,*N*′-tetramethylethylenediamine
TPU	thermoplastic polyurethane
